# High HLA-F Expression Is a Poor Prognosis Factor in Patients with Nasopharyngeal Carcinoma

**DOI:** 10.1155/2018/7691704

**Published:** 2018-10-30

**Authors:** Bo Wu, Haihua Yang, Shenpeng Ying, Hongsheng Lu, Wei Wang, Jiaming Lv, Huacai Xiong, Wei Hu

**Affiliations:** ^1^The Department of Radiotherapy, Taizhou Central Hospital (Taizhou University Hospital), Taizhou, Zhejiang, China; ^2^The Department of Radiotherapy, Taizhou Hospital of Zhejiang Province, Linhai, Zhejiang, China

## Abstract

**Background and Aims:**

In patients with nasopharyngeal carcinoma (NPC), local treatment failure and distant metastasis contribute largely to poor outcomes. The nasopharynx is an important lymphoid tissue, and NPC tumourigenesis and development are partly attributed to immune system disorders. Human leukocyte antigen F (HLA-F) has shown a close correlation with NPC in many genome-wide association studies (GWASs). However, clinical studies rarely explore the relationship of HLA-F expression with the clinical parameters and outcomes in patients with NPC.

**Methods:**

In this study, we used immunohistochemistry to evaluate HLA-F expression in 74 paraffin-embedded NPC tissue sections and then analysed the association between HLA-F expression and clinical parameters and outcomes. The plasma concentration of soluble HLA-F (sHLA-F) in NPC patients and normal controls was also detected, via enzyme-linked immunosorbent assay (ELISA).

**Results:**

Low, moderate, and high HLA-F expression levels were observed in 47.3% (35/74), 35.1% (26/74), and 17.6% (13/74), respectively, of the tissue samples. HLA-F expression showed a significant correlation with local recurrence (*p* = 0.037) and distant metastasis (*p* = 0.024) and was also an independent factor for local recurrence-free survival (LRFS; *p* = 0.016) and distant metastasis-free survival (DMFS; *p* = 0.004). Although the mean concentration of plasma sHLA-F in the NPC patients was higher than that in the normal controls (13.63 pg/ml vs. 10.06 pg/ml), no statistical significance was observed (*p* = 0.118).

**Conclusions:**

Our study provides the first evidence that high HLA-F expression is associated with NPC local recurrence and distant metastasis and may be regarded as a poor prognostic factor for NPC patients. Additional studies using larger sample sizes may be necessary to determine whether sHLA-F is a feasible NPC diagnostic indicator.

## 1. Introduction

Nasopharyngeal carcinoma (NPC) is a common malignant tumour in Southern China [1]. An estimated 42,100 new cases and 21,320 deaths were attributed to NPC in China in 2013, accounting for 1.14% of all new cancer cases and 0.96% of all cancer-related deaths [2]. The nasopharynx was identified as an important lymphoid tissue several decades ago [3]. NPC is an Epstein-Barr virus-infected malignant tumour, and failed local treatment and distant metastasis largely contribute to the poor outcome that NPC patients exhibit [4, 5]. NPC is frequently regarded as an immunity-associated malignant tumour, as NPC tumourigenesis and development are partly attributed to immune system disorders [6].

Human leukocyte antigen (HLA) is encoded by the major histocompatibility complex (MHC) I and II genes. MHC class I molecules comprise the classical (class Ia; HLA-A, HLA-B, and HLA-C) and the nonclassical (class Ib; HLA-E, HLA-F, HLA-G, and HLA-H) high-iron (Fe) molecules (HFE molecules). Aberrant expression of nonclassical HLA Ib antigens on tumour cells may mediate evasion from antitumour immune responses and have a negative clinical impact [7, 8]. A genome-wide association study (GWAS) has suggested that multiple genes in the HLA regions play important roles in the development of infectious tumours and other immunity diseases such as NPC [9].

HLA-F was discovered in chromosome 6p21.3 by Geraghty et al. in 1990 and has little allelic polymorphism with a conserved function [10, 11]. Accumulative clinical evidence has confirmed that HLA-F expression is associated with the clinical parameters and outcomes of several malignant tumours, such as breast cancer, gastric cancer, neuroblastoma, and hepatocellular carcinoma [12–15]. Four GWASs have revealed a close correlation between the HLA-F gene and NPC [16–19]. Therefore, alterations in HLA-F expression are thought to play a critical role in NPC progression. However, clinical studies to explore the relationship of HLA-F expression with the clinical parameters and outcomes in patients with NPC have rarely been conducted.

In this study, we detected HLA-F expression with immunohistochemistry in NPC lesions and then evaluated its correlation with clinical parameters and outcomes in NPC patients. In addition, we also compared the plasma levels of soluble HLA-F (sHLA-F) between NPC patients and healthy volunteers.

## 2. Materials and Methods

### 2.1. Ethical Approval

The study was approved by the Institutional Review Board committee of Taizhou Central Hospital (Taizhou University Hospital) and then performed in accordance with the ethical standards of the Helsinki Declaration and its later amendments. All participants signed an informed consent form before enrollment.

### 2.2. Tissue Samples

In this study, seventy-four primary NPC lesions were collected at Taizhou Hospital of Zhejiang Province between July 2007 and June 2015. In addition, forty chronic nasopharyngitis lesions were also obtained from March to June 2015. All the patients were pathologically diagnosed with NPC or chronic nasopharyngitis via immunohistochemistry. None of the NPC patients had distant metastasis and none of them received radiotherapy, chemotherapy, or other medical interventions before the enrollment; there was no occurrence of a second primary tumour during the follow-up period. Chronic nasopharyngitis patients with another primary tumour or severe infectious diseases were excluded. The tumour-node-metastasis (TNM) staging score was determined according to the classification of malignant tumours of the 7th edition of the American Joint Committee on Cancer (AJCC) cancer staging manual [20]. There were 16 patients (21.9%) diagnosed as stage I, 8 (11.0%) as stage II, 30 (41.1%) as stage III, and 19 (26.0%) as stage IV, and one patient's TNM data was lost. No enrolled patient received radiotherapy, chemotherapy, or other medical interventions before the study, and all the enrolled NPC patients were treated after diagnosis in our hospital. There was also no previous malignant disease or a second primary tumour in any of the selected patients. The characteristics of the NPC patients are summarized in Table 1 and include the following information: age, sex, tumour differentiation, tumour stage, lymph node status, and TNM clinical stage. The average follow-up was 47.3 ± 21.8 months (range: 14–95 months).

### 2.3. Treatment Planning and Delivery

The explanation of target volume was described in our previous studies [21, 22]. Target prescription doses and critical structure limited doses were planned according to the RTOG0225 trial. Thirty-one of the enrolled 70 NPC patients received two or more simulation computed tomography scans and were then treated with replanning during intensity-modulated radiotherapy (IMRT). Notably, another 4 patients were treated with 3-dimensional conformal radiation therapy without replanning. Neoadjuvant chemotherapy was given to 21 patients prior to radiotherapy. Concurrent chemoradiotherapy with weekly paclitaxel or with paclitaxel and platinum was performed. Adjuvant chemotherapy with at least one cycle of paclitaxel and platinum was also performed for most locally advanced patients followed by radiotherapy. These chemotherapy regimens were described in our previous study [23, 24].

### 2.4. Immunohistochemistry and Staining Evaluation

The paraffin-embedded tissue blocks of NPC tissues were cut into four-micrometre-thick sections and mounted on polyline-coated slides. After deparaffinization in xylene and rehydration through a graded series of ethanol, antigen retrieval was performed at 100°C for 5 min in 0.01 M sodium citrate buffer (pH 6.0). Endogenous peroxidase activity was blocked by using a 3% hydrogen peroxide solution at room temperature for 15 min. Then, an anti-HLA-F antibody (mAb EPR6803, 1 : 300, Abcam, Cambridge, England, UK) was applied and incubated overnight at 4°C. Subsequently, a thorough washing was performed in a 0.01 M phosphate-buffered saline solution, and the primary antibody binding sites were visualized using a Good Science kit (Good Science, Shanghai, China) according to the manufacturer's instructions. Finally, sections were counterstained with haematoxylin and mounted with glycerol gelatin.

HLA-F expression in NPC tissues was evaluated semiquantitatively by three independent pathologists without the clinical details of these patients. Membrane expression of HLA-F with or without cytoplasmic expression of HLA-F was regarded as a positive expression. HLA-F expression was graded as follows: negative (<5%), 1+ (>5–25%), 2+ (>25–50%), and 3+ (>50%). The average of three scores was calculated and confirmed by the three pathologists. In addition, negative and 1+ grades were grouped as low HLA-F expression, 2+ as moderate expression, and 3+ as high HLA-F expression.

### 2.5. sHLA-F Enzyme-Linked Immunosorbent Assay

Eighty-one NPC patients (11, 17, 39, and 14 patients for stages I, II, III, and IV, respectively) and 65 population-based, age- and sex-matched unrelated healthy volunteers were enrolled. All the NPC patients were enrolled based on the abovementioned criteria. No volunteer suffered from any primary tumour or severe infectious diseases. To protect the interest of all the patients and healthy volunteers in our study, only deidentified patient data were used. sHLA-F levels in plasma were determined with a sHLA-F-specific enzyme-linked immunosorbent assay (ELISA) kit (sHLA-F kit; TSZ, USA), according to the manufacturer's instructions. The optical density (OD) values were measured at 450 nm (Safe Heart, SHE 3000). The sHLA-F concentration was calculated via the OD value according to the standard curves, with an accuracy of 0.01 U/ml. The mean concentration for each sample analysed in triplicate was used for the subsequent analysis.

### 2.6. Statistical Analysis

Statistical analysis was performed with PASW Statistics software version 18.0. The correlations between HLA-F expression and clinical parameters were calculated with Pearson's *χ*^2^ tests, as well as the difference of HLA-F expression (positive/negative) between NPC lesions and chronic nasopharyngitis lesions. Local recurrence-free survival (LRFS), distant metastasis-free survival (DMFS), and overall survival (OS) were defined as the period from the date of diagnosis to the follow-up deadline (censored) or the date of recurrence, to the date of distant metastasis diagnosis, and to the date of death (event), respectively. Survival probabilities were calculated using the Kaplan-Meier method. Correlations between survival time and multiple clinicopathological variables were determined by univariate and multivariate analyses using Cox regression analyses. The difference in plasma sHLA-F concentration between groups was analysed with an independent-sample *t*-test and was graphed using Prism GraphPad 6. Values of *p* < 0.05 were considered significant.

## 3. Results

### 3.1. Patient Characteristics

This study comprised 52 males and 22 females, with a median age of 54 years (range: 30–82 years); 50 patients (67.6%) were diagnosed with undifferentiated carcinoma and 24 (32.4%) with differentiated carcinoma. After removing one case with no TNM stage data, 32.9% (24/73) of the patients were scored in stages I and II and 67.1% (49/73) were in stages III and IV. There were 8 patients with local recurrence and 10 with distant metastasis, and 22 died during the follow-up period. Detailed clinical characters are shown in Table 1.

### 3.2. Expression of HLA-F in NPC Lesions and Chronic Nasopharyngitis Lesions

**Figure 1:**
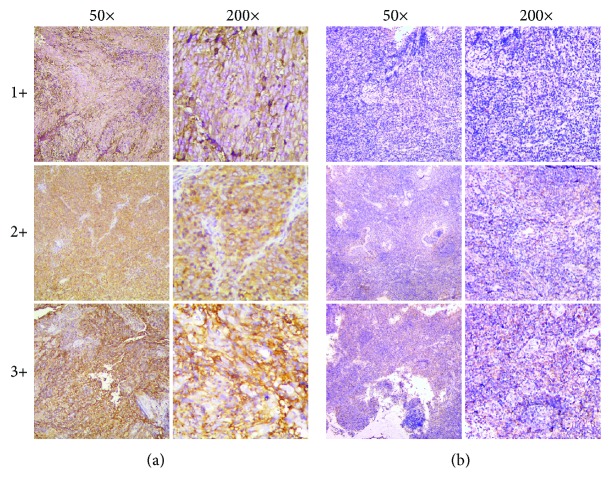
Immunohistochemical staining of HLA-F expression in primary NPC lesions and chronic nasopharyngitis lesions: (a) different expression levels (1+, 2+, and 3+) of HLA-F under 50x and 200x magnification in NPC lesions; (b) different expression levels (1+, 2+, and 3+) of HLA-F under 50x and 200x magnification in chronic nasopharyngitis lesions.

Positive staining was observed in the membrane and cytoplasmic regions (Figure 1). Overall, HLA-F expression was observed in 63 of 74 (85.1%) NPC and 13 of 40 (32.5%) chronic nasopharyngitis paraffin-embedded tissue sections. A statistical significance of HLA-F-positive/-negative expression was observed between NPC and chronic nasopharyngitis lesions (*p* = 0.000). Among the HLA-F-positive lesions of NPC, HLA-F expression was graded as 1+ in 38.1% (24/63) of the samples, as 2+ in 41.3% (26/63), and as 3+ in 20.6% (13/63). According to the group definitions, there were 47.3% (35/74), 35.1% (26/74), and 17.6% (13/74) of the samples classified as having low, moderate, or high HLA-F expression, respectively (Table 1).

### 3.3. Association of HLA-F Expression with Clinicopathological Parameters

To identify the clinical relevance of HLA-F expression in NPC, the correlations were examined between HLA-F expression and clinicopathological parameters such as sex, age, tumour differentiation, tumour stage, lymph node status, TNM stage, disease recurrence, and distant metastasis. HLA-F expression in NPC lesions was significantly associated with disease recurrence (*p* = 0.037) and distant metastasis (*p* = 0.024). No significant associations were observed between HLA-F expression and sex, age, differentiation, tumour stages T1 + T2 or T3 + T4, lymph node status N0 or N1–3, or TNM stages I + II or III + IV (Table 1).

### 3.4. Association of HLA-F Expression with Local Recurrence-Free Survival, Distant Metastasis-Free Survival, and Overall Survival

**Figure 2:**
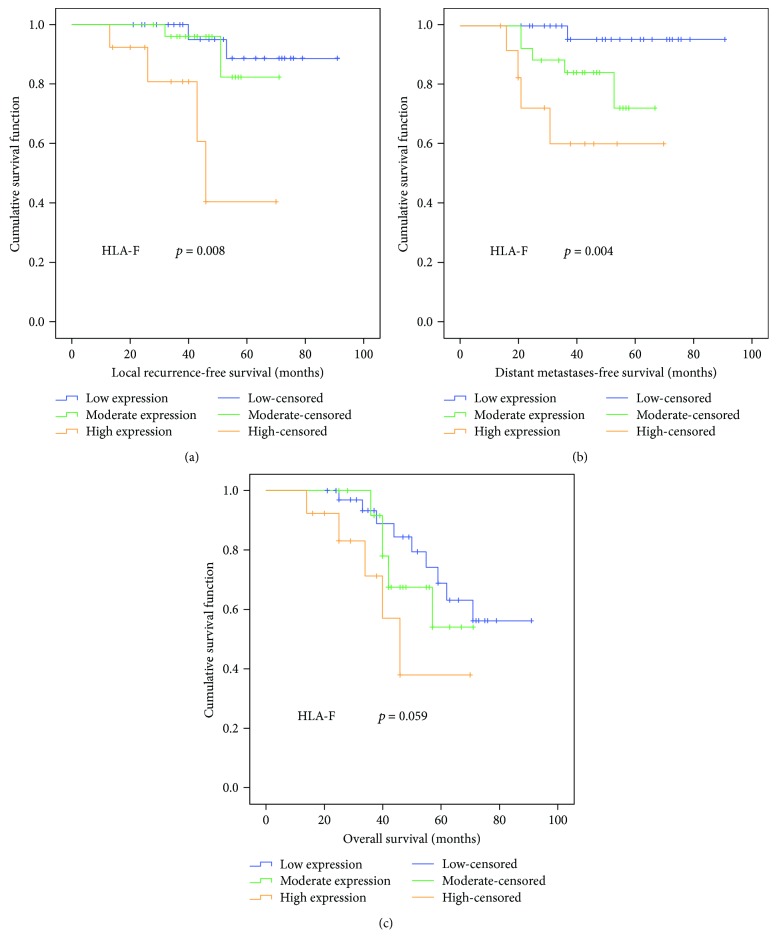
Kaplan-Meier survival analyses of NPC patients: (a) comparison of local recurrence-free survival (LRFS) among patients with low, moderate, and high HLA-F expression (*p* = 0.020); (b) comparison of distant metastasis-free survival (DMFS) among patients with low, moderate, and high HLA-F expression (*p* < 0.001); (c) comparison of overall survival (OS) among patients with low, moderate, and high HLA-F expression (*p* = 0.293).

The relationship of HLA-F expression with clinicopathological parameters of LRFS, DMFS, and OS was further investigated in NPC patients. Univariate and multivariate Cox proportional regression analyses showed that HLA-F expression (hazard ratio: 3.87; 95% confidence interval (CI): 1.29–11.58; *p* = 0.016) and tumour stage (hazard ratio: 4.31; 95% CI: 1.01–18.46; *p* = 0.049) were independent factors for LRFS (Table 2, Figure 2(a)). Similarly, HLA-F expression (hazard ratio: 3.65; 95% CI: 1.53–8.73; *p* = 0.004) was found to be an independent factor for DMFS (Table 3, Figure 2(b)). However, HLA-F expression was not an independent prognostic predictor for OS (*p* = 0.059; Table 4, Figure 2(c)).

### 3.5. Plasma sHLA-F Expression in NPC Patients

**Figure 3:**
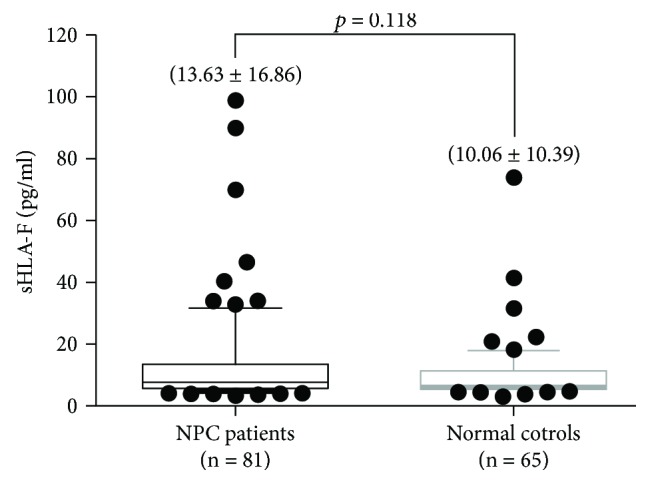
Comparison of plasma sHLA-F concentrations between the NPC patients and the normal controls (*p* = 0.118).

Plasma samples of 81 NPC patients and 65 healthy volunteers were collected, and plasma sHLA-F expression levels were detected using ELISA. The mean concentration of plasma sHLA-F was 13.63 pg/ml (ranging from 3.39 to 98.91) in NPC patients and was higher than 10.06 pg/ml (ranging from 3.22 to 74.05 pg/ml) in normal controls. However, data analysis showed that no statistically significant difference was observed in sHLA-F expression levels between the NPC patients and the normal controls (*p* = 0.118; Figure 3).

## 4. Discussion

The nasopharynx is an important lymphoid tissue [3], and NPC is regarded as an immunity-associated malignant tumour because NPC tumourigenesis and development are partly attributed to immune system disorders [6]. Among the mechanisms by which NPC cells evade the host anticancer immune system, the HLA gene and protein expression have been an issue of focus [7, 25]. A GWAS suggests that multiple genes in the HLA regions may play an important role in the development of infectious tumours including NPC [9].

HLA-F, a nonclassical HLA Ib molecule, showed a probable association with NPC tumourigenesis and development in a series of GWASs [16–19]. A meta-analysis involving single-nucleotide polymorphisms showed that HLA-F (rs3129055, T>C) was associated with increased susceptibility to NPC [26]. In addition, radiation resistance frequently leads to tumour recurrence and metastasis including NPC, which was involved in nonclassical HLA-I molecules [27]. Li et al. analysed and showed a HLA-F mRNA upregulation in the radioresistant NPC cell lines CNE-2Rs and 6-10B-Rs [28]. Therefore, we believe that HLA-F expression may influence the prognosis of NPC patients.

In our study, there was a higher proportion of HLA-F-positive expression in NPC patients (63/74) compared to chronic nasopharyngitis patients (13/40) with a significant difference (*p* = 0.000). HLA-F expression showed significant correlation with local recurrence (*p* = 0.037) and distant metastasis (*p* = 0.024). For NPC patients, the univariate analysis revealed that overexpression of HLA-F was correlated with poor LRFS (*p* = 0.008) and DMFS (*p* = 0.004) but not with OS (*p* = 0.059). Furthermore, the multivariate analysis demonstrated that HLA-F expression was an independent prognostic factor for LRFS (*p* = 0.016) and DMFS (*p* = 0.004). Similar results have been observed in other malignant tumours. Xu et al. detected HLA-F expression of primary hepatocellular carcinoma (HCC) and showed that positive HLA-F expression was observed in 47.8% (43/90) of HCC lesions and in 10.9% (6/55) of the normal liver tissues [12]. Patients with HLA-F-positive tumours had worse survival than those with HLA-F-negative tumours (*p* = 0.04). Harada et al. detected cancerous HLA-F (91 cases) and stromal HLA-F-positive infiltrating cells (186 cases) in breast cancer patients [13]. The HLA-F-positive group showed significantly poorer outcomes than the HLA-F-negative group in stage II breast cancer (*p* < 0.05). Ishigami et al. reported 209 patients with gastric cancer and demonstrated a significantly poorer five-year survival rate in the patients of HLA-F-positive expression than in those of HLA-F-negative expression [14]. However, Melsted et al. enrolled 200 patients with primary cutaneous melanoma, and HLA-F was not indicative of the prognosis [29]. In our study, patients with upregulated HLA-F expression had significantly worse survival than those with unchanged and downregulated HLA-F expression. No study on HLA-F expression has focused on NPC, but our results provide new evidence for the correlation of HLA-F expression and NPC prognosis.

The correlation analyses of HLA-F expression with the clinical parameters of NPC patients inspired us to determine whether the plasma sHLA-F expression in NPC patients was different from that in normal controls. We, thus, evaluated the sHLA-F expression level in plasma samples from 81 NPC patients and 65 healthy volunteers. Although no statistical significance was observed, the mean, minimum, and maximum concentrations of plasma sHLA-F in NPC patients were higher than those in normal controls (*p* = 0.118; Figure 3). Morandi et al. detected plasma levels of sHLA-F in neuroblastoma (NB) patients and healthy children and found that the sHLA-F levels were different between patients with metastatic and localized tumours [15]. sHLA-F was significantly associated with worse event-free survival (EFS)/OS in the whole cohort of NB patients and in patients with metastatic NB. mRNA detection of HLA-F adjacent transcript 10 (FAT10), a member of a ubiquitin-like protein family, showed higher positive rates in HCC and colon cancer patients than in the controls, meaning that FAT10 mRNA is a promising serological marker for HCC and CC [30]. Therefore, further study on the diagnosis and prognosis of NPC should focus on HLA-F and its downstream molecules.

High incidences of local recurrence and distant metastasis were observed. Several probable causes should be focused on. In our study, 67.12% (49/73) of locally advanced patients and 36.98% (27/73) of patients with tumour stage T3 or T4 NPC experienced local recurrence and/or distant metastasis. Although radiotherapy was performed in all NPC patients, some patients did not receive systematic and integrated concurrent chemotherapy with radiotherapy or subsequent adjuvant chemotherapy because of serious adverse events. Due to limited samples, no RNA expression was detected. In addition, plasma specimens analysed for sHLA-F expression were not collected from the enrolled NPC patients from which the tissue samples were obtained for HLA-F immunohistochemistry; therefore, we cannot determine whether sHLA-F is a feasible diagnostic indicator for NPC patients. Therefore, we are collecting sufficient paraffin-embedded tissue blocks and plasma specimens from the same NPC patients for further analysis.

## Figures and Tables

**Table 1:** Association of HLA-F expression in NPC lesions with clinicopathological parameters.

Characteristics	*N*	HLA-F expression			
		Low	Moderate	High	*p* value
		(*N* = 35)	(*N* = 26)	(*N* = 13)	
*Gender*					
Male	52	27	17	8	0.458
Female	22	8	9	5	
*Age (years)*					
<54	37	16	12	9	0.311
≥54	37	19	14	4	
*Tumour differentiation*					
Undifferentiated	50	24	15	11	0.235
Differentiated	24	11	11	2	
*Tumour stage*					
T1 + T2	46	25	14	7	0.221
T3 + T4	27	9	12	6	
*Lymph node status*					
N0	21	12	7	2	0.389
N1–3	52	22	19	11	
*TNM clinical stage*					
I + II	24	14	5	5	0.179
III + IV	49	20	21	8	
*Local recurrence*					
No	66	33	24	9	0.037
Yes	8	2	2	4	
*Distant metastasis*					
No	64	34	21	9	0.024
Yes	10	1	5	4	

Abbreviation: HLA-F: human leukocyte antigen F; TNM: tumour node metastasis.

**Table 2:** Cox proportional regression analyses of variables affecting the local recurrence-free survival (LRFS) in NPC patients.

Variables	Subsets	Local recurrence-free survival			
		Univariate analysis		Multivariate analysis	
		HR (95% CI)	*p* value	HR (95% CI)	*p* value
HLA-F expression	High vs. low and moderate	3.80 (1.43–10.11)	0.008	3.87 (1.29–11.58)	0.016
Gender	Male vs. female	0.41 (0.10–1.66)	0.213		
Age (years)	<54 vs. ≥54	0.29 (0.06–1.46)	0.133		
Tumour differentiation	Undifferentiated vs. differentiated	2.01 (0.50–8.07)	0.325		
Tumour stage	T1 + T2 vs. T3 + T4	5.36 (1.25–23.06)	0.024	4.31 (1.01–18.46)	0.049
Lymph node status	N0 vs. N1–3	3.04 (0.37–24.72)	0.299		
TNM stage	I + II vs. III + IV	1.76 (0.35–8.75)	0.490		

Abbreviation: HR: hazard ratio; CI: confidence interval; HLA-F: human leukocyte antigen F; TNM: tumour node metastasis.

**Table 3:** Cox proportional regression analyses of variables affecting the distant metastasis-free survival (DMFS) in NPC patients.

Variables	Subsets	Distant metastasis-free survival			
		Univariate analysis		Multivariate analysis	
		HR (95% CI)	*p* value	HR (95% CI)	*p* value
HLA-F expression	High vs. low and moderate	3.65 (1.53–8.73)	0.004	3.65 (1.53–8.73)	0.004
Gender	Male vs. female	0.90 (0.23–3.50)	0.882		
Age (years)	<54 vs. ≥54	3.96 (0.84–18.66)	0.082		
Tumour differentiation	Undifferentiated vs. differentiated	0.49 (0.10–2.31)	0.367		
Tumour stage	T1 + T2 vs. T3 + T4	1.49 (0.42–5.35)	0.541		
Lymph node status	N0 vs. N1–3	1.58 (0.33–7.43)	0.565		
TNM stage	I + II vs. III + IV	4.81 (0.61–38.03)	0.136		

Abbreviation: HR: hazard ratio; CI: confidence interval; HLA-F: human leukocyte antigen F; TNM: tumour node metastasis.

**Table 4:** Cox proportional regression analyses of variables affecting the overall survival (OS) in NPC patients.

Variables	Subsets	Overall survival			
		Univariate analysis		Multivariate analysis	
		HR (95% CI)	*p* value	HR (95% CI)	*p* value
HLA-F expression	High vs. low and moderate	1.76 (0.98–3.16)	0.059		
Gender	Male vs. female	0.93 (0.38–2.27)	0.864		
Age (years)	<54 vs. ≥54	2.03 (0.83–4.99)	0.122		
Tumour differentiation	Undifferentiated vs. differentiated	0.69 (0.27–1.77)	0.443		
Tumour stage	T1 + T2 vs. T3 + T4	2.21 (0.91–5.36)	0.080		
Lymph node status	N0 vs. N1–3	1.90 (0.64–5.63)	0.250		
TNM stage	I + II vs. III + IV	6.25 (1.45–27.01)	0.014	4.91 (1.11–21.82)	0.037
Local recurrence	No vs. yes	1.31 (0.38–4.44)	0.668		
Distant metastasis	No vs. yes	4.34 (1.73–10.91)	0.002	3.84 (1.17–7.59)	0.022

Abbreviation: HR: hazard ratio; CI: confidence interval; HLA-F: human leukocyte antigen F; TNM: tumour node metastasis.

## Data Availability

The data used to support the findings of this study are available from the corresponding author upon request.
